# Development of a gastric cancer risk calculator for questionnaire-based surveillance of Iranian dyspeptic patients

**DOI:** 10.1186/s12876-024-03123-z

**Published:** 2024-01-18

**Authors:** Kimiya Gohari, Samaneh Saberi, Maryam Esmaieli, Mohammad Tashakoripour, Mahmoud Eshagh Hosseini, Azin Nahvijou, Mohammad Ali Mohagheghi, Anoshirvan Kazemnejad, Marjan Mohammadi

**Affiliations:** 1 HPGC Research Group, Department of Medical Biotechnology, Biotechnology Research Center, Pasteur Institute of Iran, Tehran, Iran; 2 Department of Biostatistics, Faculty of Medical Sciences, Tarbiat Modares University, Tehran, Iran; 3 Gastroenterology Department, Amiralam Hospital, Tehran University of Medical Sciences, Tehran, Iran; 4 Cancer Research Center, Cancer Institute, Tehran University of Medical Sciences, Tehran, Iran

**Keywords:** Gastric cancer, Nonulcer dyspepsia, Risk prediction, Calculator, Logistic regression

## Abstract

**Background:**

Gastric cancer (GC) is considered a silent killer, taking more than three quarters of a million lives annually. Therefore, prior to further costly and invasive diagnostic approaches, an initial GC risk screening is desperately in demand.

**Methods:**

In order to develop a simple risk scoring system, the demographic and lifestyle indices from 858 GC and 1132 non-ulcer dyspeptic (NUD) patients were analysed. We applied a multivariate logistic regression approach to identify the association between our target predictors and GC *versus* NUD. The model performance in classification was assessed by receiver operating characteristic (ROC) analysis. Our questionnaire covering 64 predictors, included known risk factors, such as demographic features, dietary habits, self-reported medical status, narcotics use, and SES indicators.

**Results:**

Our model segregated GC from NUD patients with the sensitivity, specificity, and accuracy rates of 85.89, 63.9, and 73.03%, respectively, which was confirmed in the development dataset (AUC equal to 86.37%, *P* < 0.0001). Predictors which contributed most to our GC risk calculator, based on risk scores (RS) and shared percentages (SP), included: 1) older age group [> 70 (RS:+ 241, SP:7.23), 60–70 (RS:+ 221, SP:6.60), 50–60 (RS:+ 134, SP:4.02), 2) history of gastrointestinal cancers (RS:+ 173, SP:5.19), 3) male gender (RS:+ 119, SP:3.55), 4) non-Fars ethnicity (RS:+ 89, SP:2.66), 5) illiteracy of both parents (RS:+ 78, SP:2.38), 6) rural residence (RS:+ 77, SP:2.3), and modifiable dietary behaviors (RS:+ 32 to + 53, SP:0.96 to 1.58).

**Conclusion:**

Our developed risk calculator provides a primary screening step, prior to the subsequent costly and invasive measures. Furthermore, public awareness regarding modifiable risk predictors may encourage and promote lifestyle adjustments and healthy behaviours.

**Supplementary Information:**

The online version contains supplementary material available at 10.1186/s12876-024-03123-z.

## Introduction

According to the International Agency for Research on Cancer (IARC), gastric cancer (GC) is responsible for more than 769,000 global deaths, equating to one in every 13 deaths, for the year 2020 [1]. GC is more prevalent amongst the male population, such that ~ 49 in 100,000 males suffer from this disease, which is more than twice its prevalence in females (~ 21 in 100,000) [2]. Stomach cancer mainly involves older people, with the average age of diagnosis being 68 and more than half of people diagnosed are 65 or older [3].

GC is a multistep and multifactorial process involving genetic and environmental factors [4]. Besides age and gender as known risk factors, there is much evidence that unhealthy diets [5, 6], alcohol abuse [7], smoking [8–10] and other factors such as genetics, environmental and behavioural factors [11–14] enhance the risk of GC development.

Considering the incidence rates of GC in most countries are expected to decrease through 2030, reductions in smoking, prevalence of *Helicobacter pylori* infection and diet improvement will be the likely contributing factors [15]. 

It is most desired to estimate the primary disease risk using general information, consuming the least time and resources [16]. This can be made possible by combining clinical knowledge with applied data science [17]. The ultimate product should be an optimal tool, readily performed by anyone without expert knowledge, using their personal information. Ideally, the application of such tools will help increase awareness and ultimately reduce the burden of disease on the community and the health care system [18].

Classification methods are usually used to develop a risk score and identify high-risk individuals in a population [19–22]. In this study, we have used multivariate analysis to identify individual predictors differentiating GC from NUD patients. For this purpose, we carried out a logistic regression approach, using 64 predictors of known risk factors including demographic features, dietary habits, self-reported medical status, narcotics use, and SES indicators. Developing a time and cost-effective algorithm that uses the personal medical history and lifestyle habits to screen subjects for GC risk, can provide a tool for filtering dyspeptic patients prior to the more invasive screening approaches.

## Materials and methods

### Study setting

This hospital-based observational study was conducted on a group of Iranian gastric cancer (GC) patients (*n* = 858), who were consecutively (July 2003 to Jan 2020) referred to the National cancer Institute of Iran (NCII). Our GC cases were diagnosed with histologically confirmed gastric adenocarcinoma. The non-ulcer dyspeptic (NUD) patients (*n* = 1132) were those who had referred for upper gastroscopy, but lacked GC. NUD patients were admitted at the endoscopy unit of Amiralam Hospital. Both centers shared similar SES profiles. The anatomic location (subsite) of the tumor was classified as cardia (defined as cardioesophageal junction, oesophagogastric junction and gastroesophageal junction) or non-cardia (all other locations in the stomach) [23]. Histopathologic studies identified the subtype of the gastric tumors, as intestinal or diffuse [24].

Trained technicians interviewed each participant at the time of recruitment, using a structured questionnaire. This questionnaire elicited 64 predictors, including demographic features, dietary habits, self-reported medical status, narcotics use, and SES indicators. Primarily, each of the questionnaire predictors, with multiple levels (with the exception of age) was turned into binary groups (S-Table-1).

In order to use the properties of data while assuming the power of 90% for testing the significance of the odds ratio in the logistic regression model, a minimum of 936 observations were required. Therefore, our data, including 1990 observations, had sufficient power for the risk score development.

## Statistical analysis

### Imputation of the missing data

We used multivariate imputation by chained equations (MICE) [25], to deal with missing data in more than one variable. In this method, two general approaches for imputing multivariate data have been applied: joint modeling (JM) [computational strategies for multivariate linear mixed-effects models with missing values [26], multilevel models with multivariate mixed response types] and full conditional specification (FCS) [multivariate imputation by chained equations-dependency networks for inference, collaborative filtering, and data visualisation [27].

**Fig. 1:**
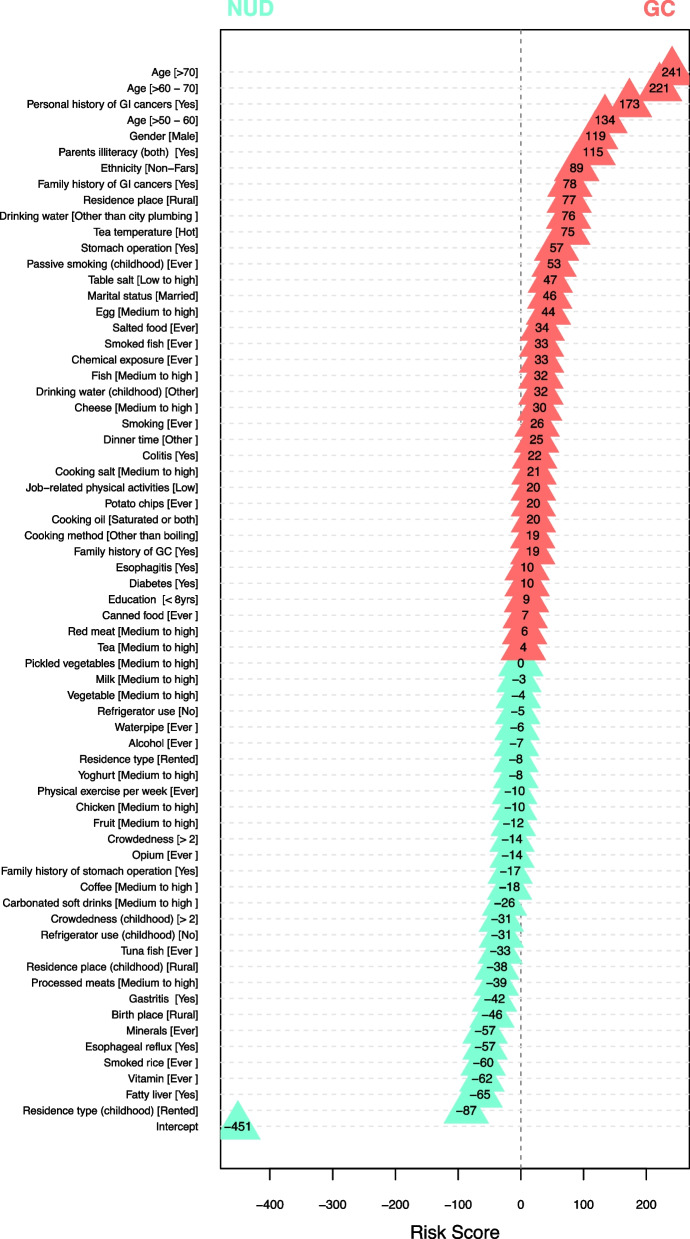
Risk score system for segregation of GC from NUD based on our logistic regression model

**Fig. 2:**
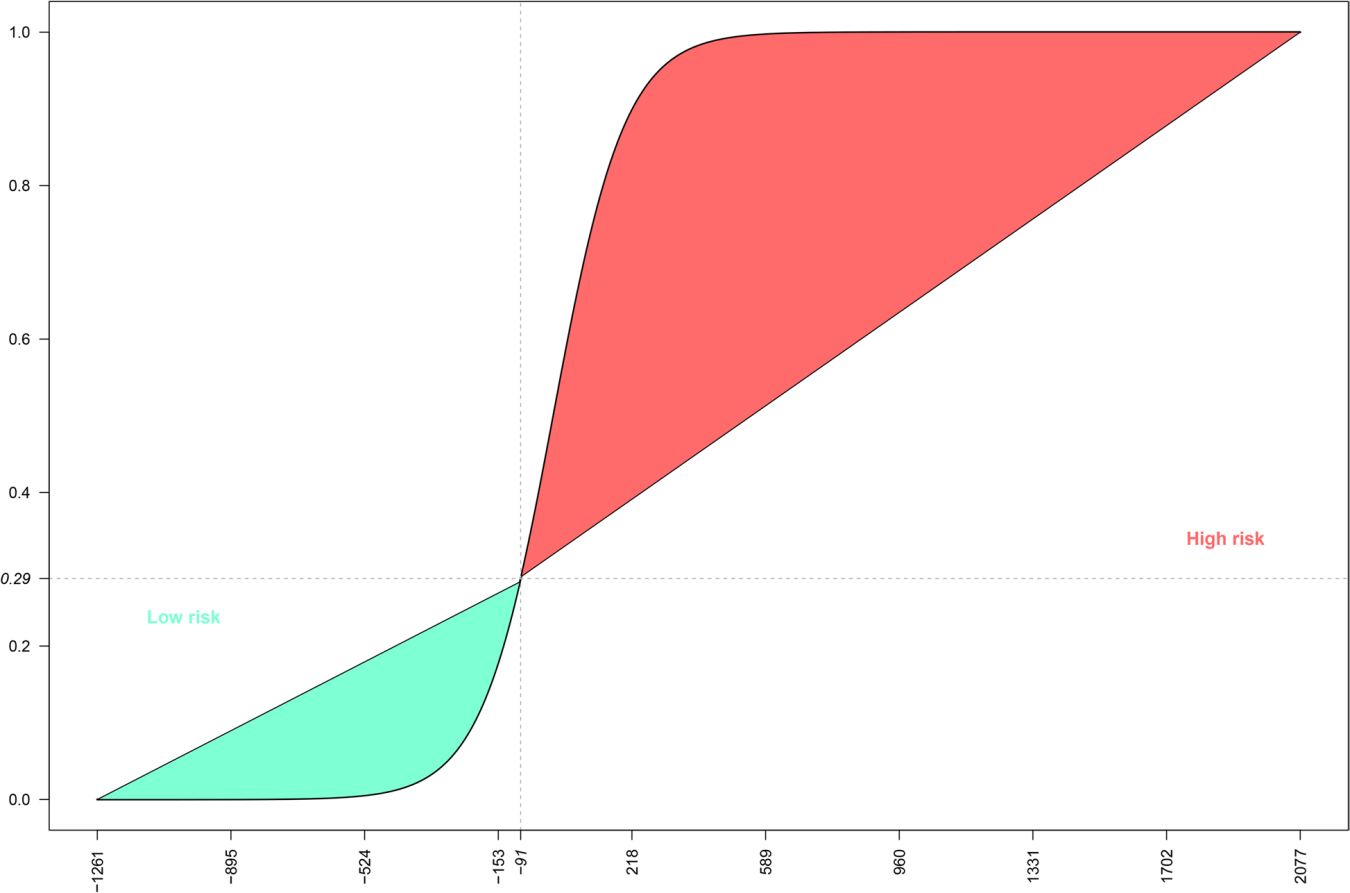
The probability of GC *versus* NUD based on risk scores

**Fig. 3:**
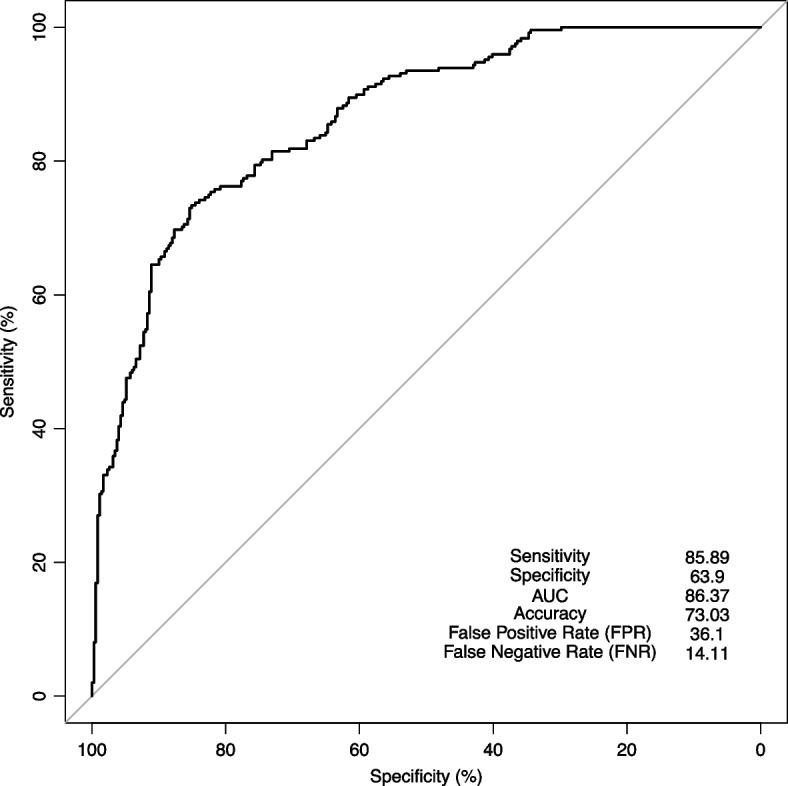
ROC curve analysis of our model differentiating GC from NUD

To validate our imputation method, we conducted the following steps. At first, using the bootstrap method [28], based on the distribution of data, we have generated ten copies of our dataset. The multivariate imputation method imputed the missing values in each copy, and five new complete datasets were generated for all the copies. In this manner, we achieved 50 complete datasets. The distribution of all variables in the original dataset and these 50 imputed versions were compared. The variables were kept in the dataset, if the deviation in the mean (S-Fig. 1) and standard deviation (S-Fig. 2) did not exceed 0.05. Next, we randomly converted 10% of the observed values for each variable into missing and imputed them again. This process was repeated 1000 times and all the variables’ biases were calculated (S-Fig. 3). The cut-off value for the bias variation was set at 2%. We aimed to maintain the imputation bias under this cut-off value.

### Model development

We used Chi-square test to measure associations between predictors and outcomes (Table 1). Statistical significance was determined using 2-sided *P*-values, with values < 0.05 considered as statistically significant. We have presented a univariate analysis of all predictors and assessed the association between each of them with GC *vs.* NUD, without taking into consideration the other predictors. In this step, we emphasized on the distribution of each (Table 1). In the next step we performed multivariate logistic regression analysis on 70% of randomly selected observations, and determined the associations with each predictor, while adjusting for all others (Table 2) [29]. The probability of having GC *vs.* NUD, based on the logistic model, was calculated [30].

**Table 1:** Distribution of predictors amongst GC *versus* NUD patients

	Predictors	levels	Overall, *N* = 1990 n (%)	GC, *N* = 858 n (%)	NUD, *N* = 1132 n (%)	*P* value^1^
**1. Demographic**						
1	Age					**< 0.001**
		≤ 50	824 (41%)	143 (17%)	681 (60%)	
		(> 50 – 60)	477 (24%)	214 (25%)	263 (23%)	
		(> 60 – 70)	431 (22%)	308 (36%)	123 (11%)	
		(> 70)	258 (13%)	193 (22%)	65 (5.7%)	
2	Ethnicity					**< 0.001**
		Fars	642 (32%)	177 (21%)	465 (41%)	
		Non-Fars	1348 (68%)	681 (79%)	667 (59%)	
3	Gender					**< 0.001**
		Female	878 (44%)	223 (26%)	655 (58%)	
		Male	1112 (56%)	635 (74%)	477 (42%)	
**2. Diet**						
4	Canned food					0.201
		Never	1317 (66%)	581 (68%)	736 (65%)	
		Ever	673 (34%)	277 (32%)	396 (35%)	
5	Carbonated soft drinks					0.212
		Never to low	1576 (79%)	690 (80%)	886 (78%)	
		Medium to high	414 (21%)	168 (20%)	246 (22%)	
6	Cheese					**0.008**
		Never to low	574 (29%)	274 (32%)	300 (27%)	
		Medium to high	1416 (71%)	584 (68%)	832 (73%)	
7	Chicken					0.732
		Never to low	1119 (56%)	486 (57%)	633 (56%)	
		Medium to high	871 (44%)	372 (43%)	499 (44%)	
8	Coffee					**0.002**
		Never to low	1937 (97%)	846 (99%)	1091 (96%)	
		Medium to high	53 (2.7%)	12 (1.4%)	41 (3.6%)	
9	Cooking method					0.401
		Boiling	811 (41%)	359 (42%)	452 (40%)	
		Other than boiling	1179 (59%)	499 (58%)	680 (60%)	
10	Cooking oil					**< 0.001**
		Unsaturated	740 (37%)	243 (28%)	497 (44%)	
		Saturated or both	1250 (63%)	615 (72%)	635 (56%)	
11	Cooking salt					**0.001**
		Never to low	626 (31%)	237 (28%)	389 (34%)	
		Medium to high	1364 (69%)	621 (72%)	743 (66%)	
12	Dinner time					**< 0.001**
		Early	1279 (64%)	495 (58%)	784 (69%)	
		Late	711 (36%)	363 (42%)	348 (31%)	
13	Drinking water (childhood)					**< 0.001**
		City plumbing	821 (41%)	221 (26%)	600 (53%)	
		Other than city plumbing	1169 (59%)	637 (74%)	532 (47%)	
14	Eggs					**< 0.001**
		Never to low	1265 (64%)	481 (56%)	784 (69%)	
		Medium to high	725 (36%)	377 (44%)	348 (31%)	
15	Fish					0.902
		Never to low	516 (26%)	224 (26%)	292 (26%)	
		Medium to high	1474 (74%)	634 (74%)	840 (74%)	
16	Fruits					**< 0.001**
		Never to low	1503 (76%)	611 (71%)	892 (79%)	
		Medium to high	487 (24%)	247 (29%)	240 (21%)	
17	Milk					**< 0.001**
		Never to low	1332 (67%)	526 (61%)	806 (71%)	
		Medium to high	658 (33%)	332 (39%)	326 (29%)	
18	Minerals					**< 0.001**
		Never	426 (21%)	114 (13%)	312 (28%)	
		Ever	1564 (79%)	744 (87%)	820 (72%)	
19	Pickled vegetables					0.245
		Never to low	414 (21%)	166 (19%)	248 (22%)	
		Medium to high	1576 (79%)	692 (81%)	884 (78%)	
20	Processed meats					**< 0.001**
		Never to low	1159 (58%)	555 (65%)	604 (53%)	
		Medium to high	831 (42%)	303 (35%)	528 (47%)	
21	Potato chips					**< 0.001**
		Never	1337 (67%)	629 (73%)	708 (63%)	
		Ever	653 (33%)	229 (27%)	424 (37%)	
22	Red meat					0.413
		Never to low	1076 (54%)	474 (55%)	602 (53%)	
		Medium to high	914 (46%)	384 (45%)	530 (47%)	
23	Salted food					0.054
		Never	1842 (93%)	783 (91%)	1059 (94%)	
		Ever	148 (7.4%)	75 (8.7%)	73 (6.4%)	
24	Smoked fish					**< 0.001**
		Never	1806 (91%)	756 (88%)	1050 (93%)	
		Ever	184 (9.2%)	102 (12%)	82 (7.2%)	
25	Smoked rice					0.611
		Never	1781 (89%)	764 (89%)	1017 (90%)	
		Ever	209 (11%)	94 (11%)	115 (10%)	
26	Table salt					**< 0.001**
		Never	1234 (62%)	450 (52%)	784 (69%)	
		Ever	756 (38%)	408 (48%)	348 (31%)	
27	Tea					**0.023**
		Never to low	53 (2.7%)	15 (1.7%)	38 (3.4%)	
		Medium to high	1937 (97%)	843 (98%)	1094 (97%)	
28	Tea temperature					**< 0.001**
		Cold to warm	1281 (64%)	450 (52%)	831 (73%)	
		Hot	709 (36%)	408 (48%)	301 (27%)	
29	Tuna fish					**< 0.001**
		Never	925 (46%)	442 (52%)	483 (43%)	
		Ever	1065 (54%)	416 (48%)	649 (57%)	
30	Vegetables					> 0.901
		Never to low	545 (27%)	236 (28%)	309 (27%)	
		Medium to high	1445 (73%)	622 (72%)	823 (73%)	
31	Vitamins					**< 0.001**
		Never	389 (20%)	93 (11%)	296 (26%)	
		Ever	1601 (80%)	765 (89%)	836 (74%)	
32	Yoghurt					**< 0.001**
		Never to low	477 (24%)	159 (19%)	318 (28%)	
		Medium to high	1513 (76%)	699 (81%)	814 (72%)	
**3. Medical status (self-reported)**						
33	Colitis					0.825
		No	1968 (99%)	849 (99%)	1119 (99%)	
		Yes	22 (1.1%)	9 (1.0%)	13 (1.1%)	
34	Diabetes					> 0.932
		No	1850 (93%)	797 (93%)	1053 (93%)	
		Yes	140 (7.0%)	61 (7.1%)	79 (7.0%)	
35	Esophageal reflux					**0.025**
		No	1572 (79%)	698 (81%)	874 (77%)	
		Yes	418 (21%)	160 (19%)	258 (23%)	
36	Esophagitis					0.315
		No	1976 (99%)	850 (99%)	1126 (99%)	
		Yes	14 (0.7%)	8 (0.9%)	6 (0.5%)	
37	Family history of GC					**< 0.001**
		No	1735 (87%)	712 (83%)	1023 (90%)	
		Yes	255 (13%)	146 (17%)	109 (9.6%)	
38	Family history of GI cancers					**< 0.001**
		No	1475 (74%)	585 (68%)	890 (79%)	
		Yes	515 (26%)	273 (32%)	242 (21%)	
39	Family history of stomach operation					**0.005**
		No	1832 (92%)	773 (90%)	1059 (94%)	
		Yes	158 (7.9%)	85 (9.9%)	73 (6.4%)	
40	Fatty liver					0.226
		No	1970 (99%)	852 (99%)	1118 (99%)	
		Yes	20 (1.0%)	6 (0.7%)	14 (1.2%)	
41	Gastritis					**< 0.001**
		No	1650 (83%)	758 (88%)	892 (79%)	
		Yes	340 (17%)	100 (12%)	240 (21%)	
42	Personal history of GI cancers					**< 0.001**
		No	1926 (97%)	805 (94%)	1121 (99%)	
		Yes	64 (3.2%)	53 (6.2%)	11 (1.0%)	
43	Family history of stomach operation					**< 0.001**
		No	1952 (98%)	830 (97%)	1122 (99%)	
		Yes	38 (1.9%)	28 (3.3%)	10 (0.9%)	
**4. Narcotics**						
44	Alcohol					0.415
		Never	1837 (92%)	787 (92%)	1050 (93%)	
		Ever	153 (7.7%)	71 (8.3%)	82 (7.2%)	
45	Opium					**< 0.001**
		Never	1777 (89%)	732 (85%)	1045 (92%)	
		Ever	213 (11%)	126 (15%)	87 (7.7%)	
46	Smoking					**< 0.001**
		Never	1414 (71%)	519 (60%)	895 (79%)	
		Ever	576 (29%)	339 (40%)	237 (21%)	
47	Waterpipe					0.523
		Never	1807 (91%)	775 (90%)	1032 (91%)	
		Ever	183 (9.2%)	83 (9.7%)	100 (8.8%)	
48	Passive smoking (childhood)					**< 0.001**
		Never	1235 (62%)	495 (58%)	740 (65%)	
		Ever	755 (38%)	363 (42%)	392 (35%)	
5. Socioeconomic status (SES)						
49	Birth place					**< 0.001**
		Urban	990 (50%)	332 (39%)	658 (58%)	
		Rural	1000 (50%)	526 (61%)	474 (42%)	
50	Chemical exposure					**0.003**
		Never	1704 (86%)	712 (83%)	992 (88%)	
		Ever	286 (14%)	146 (17%)	140 (12%)	
51	Crowdedness					**0.003**
		≤ 2 per room	1138 (57%)	458 (53%)	680 (60%)	
		> 2 per room	852 (43%)	400 (47%)	452 (40%)	
52	Crowdedness (childhood)					**0.003**
		≤ 2 per room	323 (16%)	115 (13%)	208 (18%)	
		> 2 per room	1667 (84%)	743 (87%)	924 (82%)	
53	Drinking water					**< 0.001**
		City plumbing	1777 (89%)	715 (83%)	1062 (94%)	
		Other than city plumbing	213 (11%)	143 (17%)	70 (6.2%)	
54	Education					**< 0.001**
		> 8 yrs	551 (28%)	128 (15%)	423 (37%)	
		< 8 yrs	1439 (72%)	730 (85%)	709 (63%)	
55	Job-related physical activities					**< 0.001**
		High	988 (50%)	500 (58%)	488 (43%)	
		Low	1002 (50%)	358 (42%)	644 (57%)	
56	Marital status					**< 0.001**
		Other	115 (5.8%)	12 (1.4%)	103 (9.1%)	
		Married	1875 (94%)	846 (99%)	1029 (91%)	
57	Parents illiteracy (both)					**< 0.001**
		No	636 (32%)	151 (18%)	485 (43%)	
		Yes	1354 (68%)	707 (82%)	647 (57%)	
58	Physical exercise per week					**< 0.001**
		Never	701 (35%)	256 (30%)	445 (39%)	
		Ever	1289 (65%)	602 (70%)	687 (61%)	
59	Refrigerator use					0.241
		Yes	1956 (98%)	840 (98%)	1116 (99%)	
		No	34 (1.7%)	18 (2.1%)	16 (1.4%)	
60	Refrigerator use (childhood)					**< 0.001**
		Yes	698 (35%)	200 (23%)	498 (44%)	
		No	1292 (65%)	658 (77%)	634 (56%)	
61	Residence place					**< 0.001**
		Urban	1617 (81%)	604 (70%)	1013 (89%)	
		Rural	373 (19%)	254 (30%)	119 (11%)	
62	Residence place (childhood)					**< 0.001**
		Urban	1073 (54%)	345 (40%)	728 (64%)	
		Rural	917 (46%)	513 (60%)	404 (36%)	
63	Residence type					**< 0.001**
		Owned	1617 (81%)	758 (88%)	859 (76%)	
		Rented	373 (19%)	100 (12%)	273 (24%)	
64	Residence type (childhood)					**< 0.001**
		Owned	1786 (90%)	807 (94%)	979 (86%)	
		Rented	204 (10%)	51 (5.9%)	153 (14%)	

^1^Pearson’s Chi-squared test

**Table 2:** The results of the multivariate logistic regression model to explore the GC-prone *versus* NUD-prone predictors

Predictors		Shared Percentages	OR (95%CI)	*P*-Value	Risk Score
**1. Demographic**					
1	Age [> 50 – 60]	4.02	3.84 (2.56, 5.74)	**< 0.001**	+134
	Age [> 60 – 70]	6.6	9.09 (5.7, 14.49)	**< 0.001**	+221
	Age [> 70]	7.23	11.18 (6.36, 19.66)	**< 0.001**	+241
2	Ethnicity [Non-Fars]	2.66	2.43 (1.74, 3.41)	**< 0.001**	+89
3	Gender [Male]	3.55	3.27 (2.03, 5.26)	**< 0.001**	+119
**2. Diet**					
4	Canned food [Ever]	0.01	1 (0.72, 1.39)	0.991	0
5	Carbonated soft drinks [Medium to high]	1	0.72 (0.49, 1.06)	0.095	−33
6	Cheese [Medium to high]	1.42	1.61 (1.15, 2.25)	**0.006**	+47
7	Chicken [Medium to high]	0.29	0.91 (0.67, 1.24)	0.548	−10
8	Coffee [Medium to high]	1.95	0.52 (0.18, 1.52)	0.231	−65
9	Cooking method [Other than boiling]	0.79	1.3 (0.95, 1.79)	0.102	+26
10	Cooking oil [Saturated or both]	0.61	1.23 (0.87, 1.72)	0.238	+20
11	Cooking salt [Medium to high]	0.61	1.23 (0.86, 1.75)	0.262	+20
12	Dinner time [Late]	1.03	1.41 (1.03, 1.93)	**0.032**	+34
13	Drinking water (childhood) [Other than city plumbing]	0.89	1.35 (0.89, 2.04)	0.161	+30
14	Eggs [Medium to high]	0.96	1.38 (1.01, 1.89)	**0.044**	+32
15	Fish [Medium to high]	0.19	1.07 (0.75, 1.51)	0.716	+6
16	Fruits [Medium to high]	0.3	0.9 (0.64, 1.28)	0.569	−10
17	Milk [Medium to high]	0.57	1.21 (0.87, 1.68)	0.259	+19
18	Minerals [Ever]	1.24	0.66 (0.43, 1.01)	0.055	−42
19	Pickled vegetables [Medium to high]	0.21	1.07 (0.74, 1.56)	0.717	+7
20	Processed meats [Medium to high]	0.22	0.93 (0.67, 1.29)	0.656	−7
21	Potato chips [Ever]	0.11	0.96 (0.68, 1.36)	0.83	−4
22	Red meat [Medium to high]	0.14	0.95 (0.7, 1.3)	0.767	−5
23	Salted food [Ever]	0.42	0.87 (0.47, 1.59)	0.646	−14
24	Smoked fish [Ever]	1.31	1.55 (0.84, 2.86)	0.161	+44
25	Smoked rice [Ever]	1.72	0.56 (0.33, 0.98)	**0.041**	−57
26	Table salt [Ever]	1.39	1.59 (1.14, 2.22)	**0.007**	+46
27	Tea [Medium to high]	0.3	1.1 (0.45, 2.7)	0.827	+10
28	Tea temperature [Hot]	1.58	1.7 (1.24, 2.33)	**0.001**	+53
29	Tuna fish [Ever]	0.94	0.73 (0.54, 0.99)	**0.044**	−31
30	Vegetables [Medium to high]	0.76	1.29 (0.9, 1.84)	0.164	+25
31	Vitamins [Ever]	1.81	0.55 (0.35, 0.85)	**0.008**	−60
32	Yogurt [Medium to high]	1.16	0.68 (0.47, 0.98)	**0.036**	−39
**3. Medical status (self-reported)**					
33	Colitis [Yes]	3.43	3.15 (0.66, 14.94)	0.148	+115
34	Diabetes [Yes]	0.24	0.92 (0.54, 1.56)	0.762	−8
35	Esophageal reflux [Yes]	0.36	0.89 (0.6, 1.3)	0.532	−12
36	Esophagitis [Yes]	0.29	1.1 (0.2, 6.14)	0.913	+10
37	Family history of GC [Yes]	0.64	1.24 (0.65, 2.36)	0.514	+21
38	Family history of GI cancers [Yes]	1.72	1.77 (1.14, 2.76)	**0.011**	+57
39	Family history of stomach operation [Yes]	0.6	1.22 (0.64, 2.35)	0.547	+20
40	Fatty liver [Yes]	2.59	0.42 (0.08, 2.3)	0.319	−87
41	Gastritis [Yes]	0.92	0.73 (0.48, 1.12)	0.151	−31
42	Personal history of GI cancers [Yes]	5.19	5.67 (1.87, 17.17)	**0.002**	+173
43	Personal history of stomach operation [Yes]	2.25	2.12 (0.64, 7.04)	0.218	+75
**4. Narcotics**					
44	Alcohol [Ever]	0.12	1.04 (0.57, 1.9)	0.895	+4
45	Opium [Ever]	0.51	0.84 (0.5, 1.42)	0.521	−17
46	Smoking [Ever]	0.99	1.39 (0.96, 2.03)	0.084	+33
47	Waterpipe [Ever]	0.66	1.25 (0.72, 2.16)	0.433	+22
48	Passive smoking (childhood) [Ever]	0.95	1.37 (1, 1.89)	0.053	+32
**5. Socioeconomic status (SES)**					
49	Birth place [Rural]	1.69	0.57 (0.36, 0.9)	**0.016**	−57
50	Chemical exposure [Ever]	0.08	0.97 (0.64, 1.49)	0.903	−3
51	Crowdedness [> 2 per room]	0.23	0.93 (0.68, 1.27)	0.634	−8
52	Crowdedness (childhood) [> 2 per room]	1.14	0.68 (0.44, 1.07)	0.096	−38
53	Drinking water [Other than city plumbing]	0.98	1.39 (0.8, 2.4)	0.243	+33
54	Education [< 8 yrs]	0.42	0.87 (0.57, 1.32)	0.51	−14
55	Job-related physical activities [Low]	0.56	1.2 (0.79, 1.85)	0.395	+19
56	Marital status [Married]	2.28	2.15 (0.95, 4.83)	0.065	+76
57	Parents illiteracy (both) [Yes]	2.35	2.19 (1.5, 3.2)	**< 0.001**	+78
58	Physical exercise per week [Ever]	0.55	0.83 (0.6, 1.16)	0.274	−18
59	Refrigerator use [No]	0.79	0.77 (0.24, 2.45)	0.655	−26
60	Refrigerator use (childhood) [No]	1.39	0.63 (0.42, 0.95)	**0.029**	−46
61	Residence place [Rural]	2.3	2.16 (1.36, 3.42)	**0.001**	+77
62	Residence place (childhood) [Rural]	0.26	1.09 (0.67, 1.79)	0.729	+9
63	Residence type [Rented]	0.19	0.94 (0.63, 1.4)	0.756	−6
64	Residence type (childhood) [Rented]	1.85	0.54 (0.32, 0.9)	**0.019**	−62
65	Intercept (other unknown factors)	13.49	0.01 (0, 0.04)	**< 0.001**	−451

The indicated levels in the parenthesis are assessed against the reference level for each predictor

The probability of being GC *versus* NUD was computed using logistic regression:

$$P(GC)=\frac{1}{1+{e}^{-\left({\beta}_0+{\beta}_1{X}_1+{\beta}_2{X}_2+\dots +{\beta}_k{X}_k\right)}}$$

Where *β*_0_ is the intercept term and *β*_1_, *β*_2_, …, *β*_*k*_ are the coefficients associated with the input features *X*_1_, *X*_2_, …, *X*_*k*_.

This study divided patients into two risk groups based on an assigned cut-off point, derived from fixing the sensitivity rate at a minimum of 90%, while maximizing the specificity rate. Accordingly, the best threshold for the risk score was identified. We defined the shared percentage for every predictor in our risk calculator, as the contribution of each variable in predicting GC *vs.* NUD, as clinical outcomes. This measure is the proportion of the standardized regression coefficient (point estimates) for each predictor relative to their total sum (Table 2 and Fig. 1). The final risk score for each predictor was calculated by the multiplication of their pertinent point estimate by 100.

### Model validation

We used the train-test split method [31] for determining the performance criteria (AUC, sensitivity, specificity, precision, false-positive, false-negative, and accuracy rates) of our logistic model (Fig. 2), as well as to assess its internal validity.

These performance criteria were calculated as follows:

The accuracy rate, which measures the overall correctness of the classification, was calculated as:

$$Accuracy=\frac{TP+ TN}{TP+ TN+ FP+ FN}$$

The sensitivity rate (true positive rate or recall), which measures the proportion of actual positive instances that were correctly identified, was calculated as:

$$Sensitivity=\frac{TP}{TP+ FN}$$

The specificity rate (true negative rate), which measures the proportion of actual negative instances that were correctly identified, was calculated as:

$$Specificity=\frac{TN}{TN+ FP}$$

Where TP is the number of true positives, TN is the number of true negatives, FP is the number of false positives and FN is the number of false negatives.

To do this, the data were randomly divided into the development (70%) and validation (30%) subsets. The performance criteria of our GC risk calculator were determined by examining calibration and discrimination measures. Calibration refers to how closely the predicted probability of having GC agrees with the observed GC status and is assessed by the Hosmer-Lemeshow test [32]. The discrimination rate expresses the ability of the model to differentiate between individuals with GC *versus* NUD. This was evaluated by calculating the area under the ROC curve (AUC) [33]. An AUC value of 50 and 100 was considered as having no *versus* perfect discrimination, respectively. Risk thresholds that gave a combination of more than 85% sensitivity rates and maximum specificity rates were derived from the list provided by the ROC curve analysis. All statistical analysis and data visualizations were done in R statistical software environment.

## Results

### Descriptive information

Our observational study included 858 GC [development = 610 and validation = 248] and 1132 NUD [development = 783 and validation = 349] patients, who were entered into this hospital-based study.

All of the 64 questionnaire predictors, with multiple levels, were converted into binary categories, as presented in S-Table 1. We have also presented the distribution of each of these predictors amongst GC *versus* NUD patients, without any adjustments for other predictors in Table 1. The results of the Chi-square test showed that the distribution of most (47/64) of the predictors were different between GC and NUD patients (Table 1). The association between predictors and GC *vs.* NUD affected the model, and although most variables were independently associated with GC (Table 1), when adjusted for all other variables, few associations, remained statistically significant (Table 2).

The data obtained from the 64 predictors from our 1990 (GC + NUD) cases included varying degrees of missingness. To remedy this, we used the MICE method to impute the missing values. But first it was critical to validate our imputation technique and ascertain a consistent distribution for each predictor thereafter. Having done so, in the first approach amongst the 50 regenerated samples, the mean (S-Fig. 1) and standard deviation (S-Figure-2) differences, between our actual and imputed data did not exceed 0.05 and were thus acceptable. In the second approach, for all 1000 bootstrap-generated samples, the bias was determined as under 0.02 (S-Fig. 3).

### Model development

Logistic regression specified the strength of association between each of our 64 predictors and the clinical outcome (GC or NUD). The strengths of association for each of the predictors (if any), while adjusting for all other predictors, are presented via risk scores, shared precents, and odds ratios (Table 2). Taking into consideration all of the 64 predictors in our model, a risk calculator was created, scoring for GC or NUD (Fig. 1). The obtained risk score ranged from − 1261 to + 2077 (total range of 3338), moving from NUD towards GC. Of this range, the risk score of − 451, equivalent to a shared percentage of 13.49, was assigned to subjects at reference level. The remaining 86.51 percentage of the risk score was contributed by our 64 predictors, with varying shares. Aiming for a minimum sensitivity rate of 90%, the risk score of − 91, coinciding with the probability value of 0.29 (ranging from 0 to 1.0, Fig. 2) was identified as the cut-off point. Keeping in mind that each of the addressed 64 predictors contributed to the final risk score, those which were statistically significant are described below.

### GC-prone predictors

The predictors which acted towards the development of GC are considered as GC-prone. Amongst the demographic category, older age holds the first place, creating risk scores of + 134 to + 221 to + 241, for subjects aged > 50 – 60, > 60 – 70 and > 70, in reference to those aged ≤ 50 years, respectively. These values were sequentially equivalent to 4.02, 6.60, and 7.23 shared percentage (SP) of the total range. Next in line, was being of male gender and non-Fars/mixed ethnicity, with risk scores of + 119 (SP = 3.55%) and + 89 (SP = 2.66%), respectively. Amongst the SES factors, the illiteracy of both parents and residence in a rural area contributed + 77 and + 78 risk scores, respectively, which contributed 2.35 and 2.3 shared percentages to the score. In regards to the medical status of the subjects, having a personal and family history of GI cancers provided a GC risk score of + 173 (SP =5.19) and + 57 (SP =1.72), respectively. Modifiable lifestyle behaviors, such as diet and use of narcotics took the subsequent positions. Amongst dietary habits, drinking hot tea [+ 53 (SP =1.58)], consumption of medium-to-high amounts of cheese [+ 47 (SP = 1.42)], use of table salt [+ 46 (SP = 1.39)], late dinnertime [+ 34 (SP = 1.03)], and consumption of medium-to-high amounts of eggs [+ 32 (S*P* = 0.96)] were amongst the dietary GC-prone predictors (Table 2).

### Association with the subtype and subsite of GC

Some of the above-mentioned GC-prone predictors were also associated with the subsite and/or histologic subtype of the tumor. Amongst these, age was closely associated with the intestinal histologic subtype of GC (*P* = 0.003). History of GI cancer was associated with the cardia anatomic location (*P* = 0.02) and intestinal histologic subtype (*P* = 0.032) of the tumor. The predictors of drinking hot tea (*P* = 0.004) and consumption of table salt (*P* = 0. 04) were associated with the cardia subset of the GC tumors.

### NUD-prone predictors

However, there were some predictors that acted towards the development of NUD; in other words, they were NUD-prone. These predictors belonged to the two categories of SES and diet. The predictors of the former category included: rented residential place (during childhood) [− 62 (SP = 1.85)], rural-birth place [− 57 (SP = 1.69)], and lacking refrigerator (during childhood) [− 46 (SP = 1.39)]. Predictors of the dietary habits included: taking vitamins [− 60 (SP = 1.81)], consumption of smoked rice [− 57 (SP = 1.72)], medium-to-high consumption of yoghurt [− 39 (SP = 1.16)], and consumption of tuna fish [− 31 (SP = 0.94)] (Table 2).

### Model validation

To validate the results of the above-described development model, a validation approach was taken, assessing 597 individuals (248 GC and 349 NUD; GC: NUD ratio, 1:1.41), on which the ROC analysis was performed (Fig. 3). Using our 64 predictors, we were able to differentiate GC from NUD, with an AUC of 86.37% and the sensitivity, specificity, and accuracy rates of 85.89, 63.9 and 73.03%, respectively. According to this model, the rates of false positives and false negatives were 36.1, and 14.11%, respectively (Fig. 3).

We have also evaluated the calibration of our model by the Hosmer-Lemeshow method. Having done so, a *P* value of 0.4761 (well above 0.05) was obtained. Thus, the fitness of our model was confirmed. Our risk calculator can, thus, calculate the risk of GC *versus* NUD, based on the probability our 64 predictors, proportional to the achieved risk score (Fig. 3).

## Discussion

Gastric cancer being a silent killer, usually catches patients and their health service providers, off-guard. Being able to assign a relative risk to subjects, based on their demographic characteristics and life style behaviours, will provide an upper hand in focusing on the at-risk subjects, with subsequent stepwise clinical testing and follow-ups. The goal of this study was to develop an approach to accomplish the primary screening step based on our target predictors.

In 2023, a multicentre population-based study, carried out on over 416 thousand subjects (aged 40 – 75 years) in China, a GC risk calculator was developed, which highlighted 11 demographic and life style variables that place individuals at risk of GC [34]. Although our study was hospital-based and has screened Iranian dyspeptic patients, the common variables between these two studies still identify age, gender (male), education (illiteracy of parents), salt intake and personal and family history of cancer as definite risk factors. In another population-based screening study on subjects (aged 40–74 years), with no history of cancer in Korea, six risk factors were identified [35], of which salt intake was a shared prominent risk factor with our hospital-based screening study.

In 2019, a population-based study was conducted in China to assess the general knowledge about GC risk factors and symptoms. The analysis was performed on 1200 adults, over the age of 18 with an average age of 40, which showed that the mean score for GC knowledge was 8.85 out of 22. Of the 1200 participants, 564 (47.0%) had insufficient understanding of GC risk factors and warning symptoms. Overall, about 84% of people believed that screening helped diagnose GC. However, only 15.2% of people were screened for GC. There were various reasons for avoiding screening, including being asymptomatic, fear of diagnostic screening and its outcomes, male gender, living in rural areas, lower educational levels, etc. [36]. Hence, lack of routine screening and the absence of specific symptoms for this fatal disease, leaves most subjects undiagnosed until the terminal stages, which accounts for GC being known as a silent killer [37, 38].

Several methodological studies on GC have been conducted over the years [13, 39–43]. A concerted strategy for the joint analysis of these investigations may allow new insights into the etiology of GC. Therefore, the ‘Stomach cancer Pooling (StoP) Project’ was set up in 2012 to join together several investigators and create a consortium of epidemiological investigations on risk factors for GC. The SToP’s final aim was to examine the role of several lifestyles and genetic determinants in the etiology of GC, through pooled analyses of individual-level data [44].

In our study we intended to investigate the effects of any potential risk factors, even if they were not statistically significant, so to create an all-inclusive risk calculator.

The GC-prone factors identified *via* our model, are also supported by previous studies, include older age [34, 45–47], male gender [34, 48], and non-Fars/mixed ethnicity [49–51], illiteracy of both parents [52–55], family history of GI cancers [56–59], drinking hot tea [60], late dinnertime [61, 62], consumption of table salt [63–65], and medium to high amounts of cheese and eggs [66–69]. Having used a logistic regression model, we have developed a gastric cancer risk calculator, with the sensitivity, specificity, and accuracy rates of 85.89, 63.9, and 73.03%, which can be used by individuals or their healthcare workers, for primary screening of dyspeptic patients.

In 2007, Driver et al. [21] developed a simple scoring system that identifies men at increased risk of colorectal cancer, based on age and modifiable behaviours, such as alcohol intake, smoking status, and body mass index. They ran a logistic regression model as well as a proportional hazards model, to better simulate a screening decision, based on the information obtained. The discrimination power of the final model was about 70% (AUC = 69.5%) [21]. In comparison, our risk score had the discrimination power (AUC) of 86.37% during internal validation. Keeping in mind that this is the primary screening step, followed by simple and complex clinical testing, the limited detection rates, we have herein obtained for a primary questionnaire-based surveillance, are acceptable.

The strengths of our study include its sensible sample size and inclusion of a wide variety of target demographic and lifestyle behaviours. However, we have used a case-case setting in order to be able to add other clinical data, on the next rounds of clinical and paraclinical screening. Having compared GC patients with non-GC (non-ulcer dyspeptic, NUD) patients, the scale bar of our risk score moves towards the direction of GC (GC-prone) or NUD (NUD-prone) and is, at best, suitable for screening dyspeptic patients, rather than the general population. Thus, our risk calculator, must be adjusted, by applying the model in a case-control (GC *versus* healthy population) setting. It must also be kept in mind that some of the highlighted risk indicators may actually be proxies for other unaddressed predictors. Furthermore, the fact that we had to turn our multinomial levels (answers), into binomial, may have oversimplified our model. Another point of concern is the external validation of this model on other sample cohorts with diverse environmental, cultural, and social characteristics.

Nevertheless, applying such an inexpensive GC risk calculator, using questionnaire-based information, can provide the first step in screening Iranian at risk patients, to be followed by more complex laboratory and clinical screenings. Furthermore, providing information about individualized GC risk status, can lead to attempts at correction of the modifiable risk behaviours. Future studies include, validation of this model in case-control settings, in different geographic locations.

### Supplementary Information

**Additional file 1.**

**Additional file 2.**

**Additional file 3.**

**Additional file 4.**

## Data Availability

The datasets used and/or analysed during the current study available from the corresponding author on reasonable request.

## Abbreviations

GC: Gastric cancer
NUD: Non-ulcer dyspeptic
ROC: Receiver operating characteristic
MOHME: Ministry of Health and Medical Education
StoP: Stomach cancer Pooling
SP: Shared percentage
FCS: Full conditional specification
JM: Joint modeling
MICE: Multivariate imputation by chained equations
NCII: National cancer institute of Iran
IARC: International Agency for Research on Cancer
RS: Risk scores
