# Supplementary figures and images for: Development of a gastric cancer risk calculator for questionnaire-based surveillance of Iranian dyspeptic patients
Source: BMC Gastroenterol. 2024 Jan 18;24:39. doi: 10.1186/s12876-024-03123-z (PMC10797901; doi:10.1186/s12876-024-03123-z)

**S-Figure 1 -** Mean differences between imputed and original data


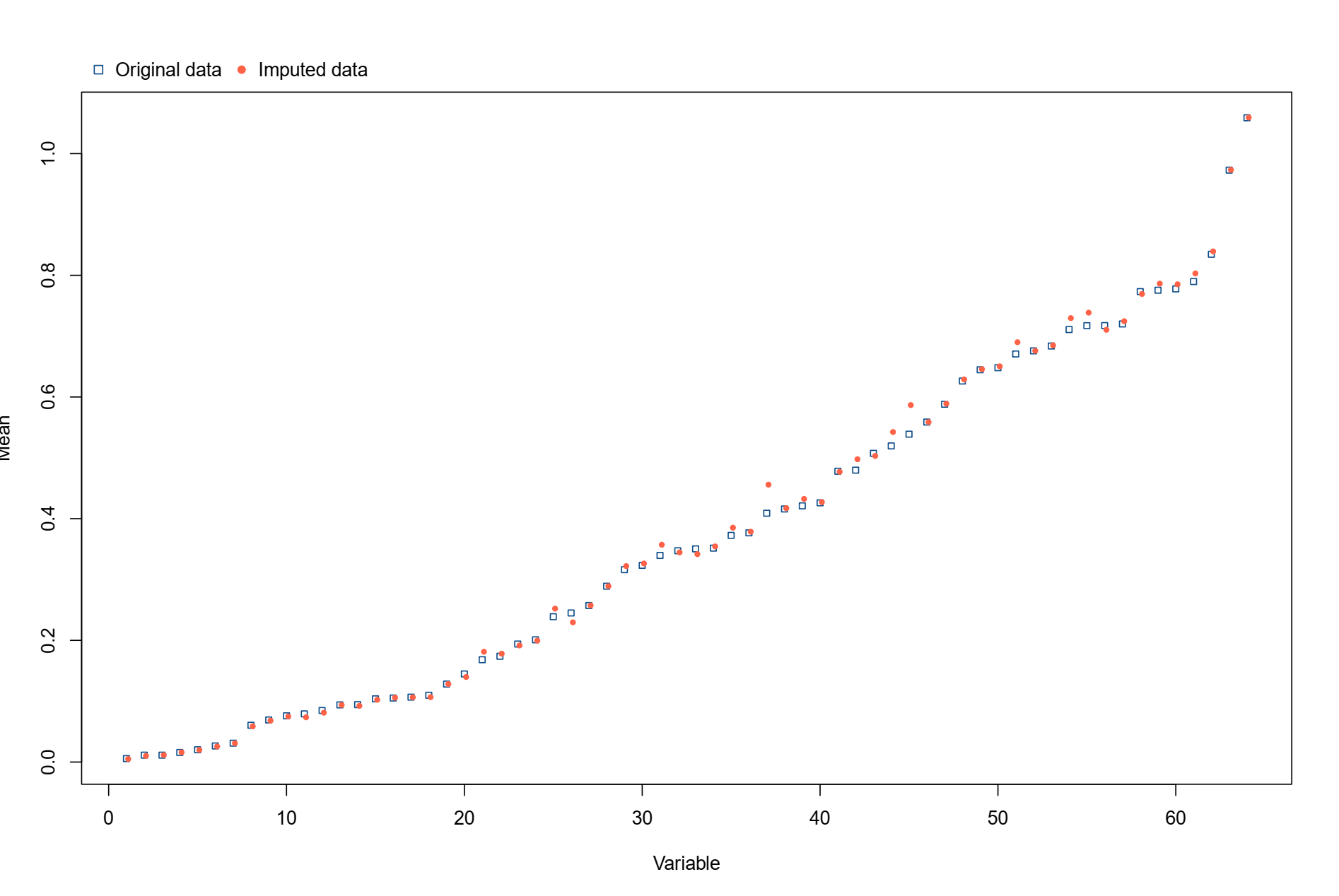

Supplement: Supplementary file 2 — Additional file 2. [file 12876_2024_3123_MOESM2_ESM.docx]

**S-Figure 2 -** Standard deviation differences between imputed and original data

**
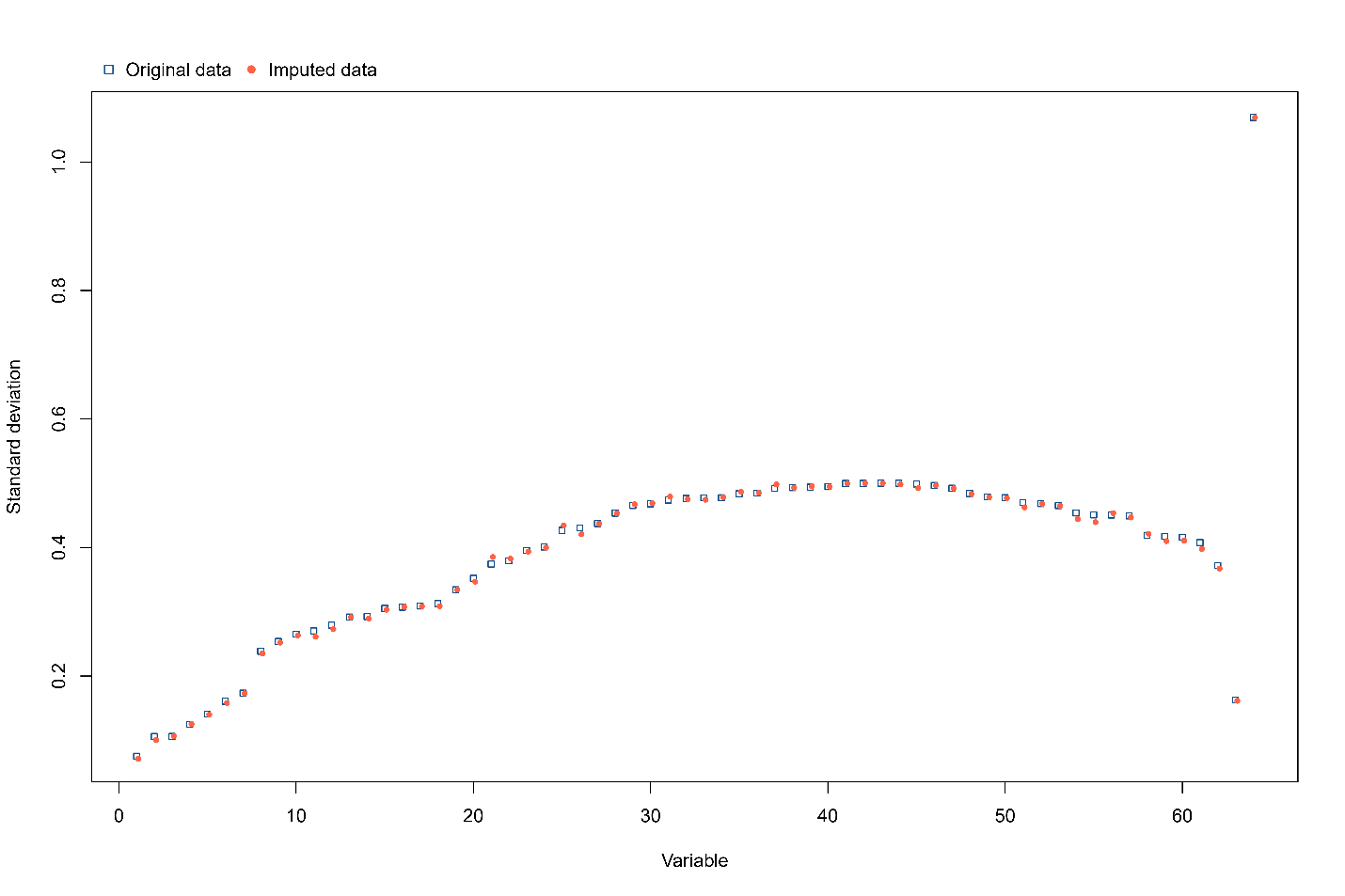
**

Supplement: Supplementary file 3 — Additional file 3. [file 12876_2024_3123_MOESM3_ESM.docx]

**S-Figure 3 - Bootstrap bias for imputation validation test**

**
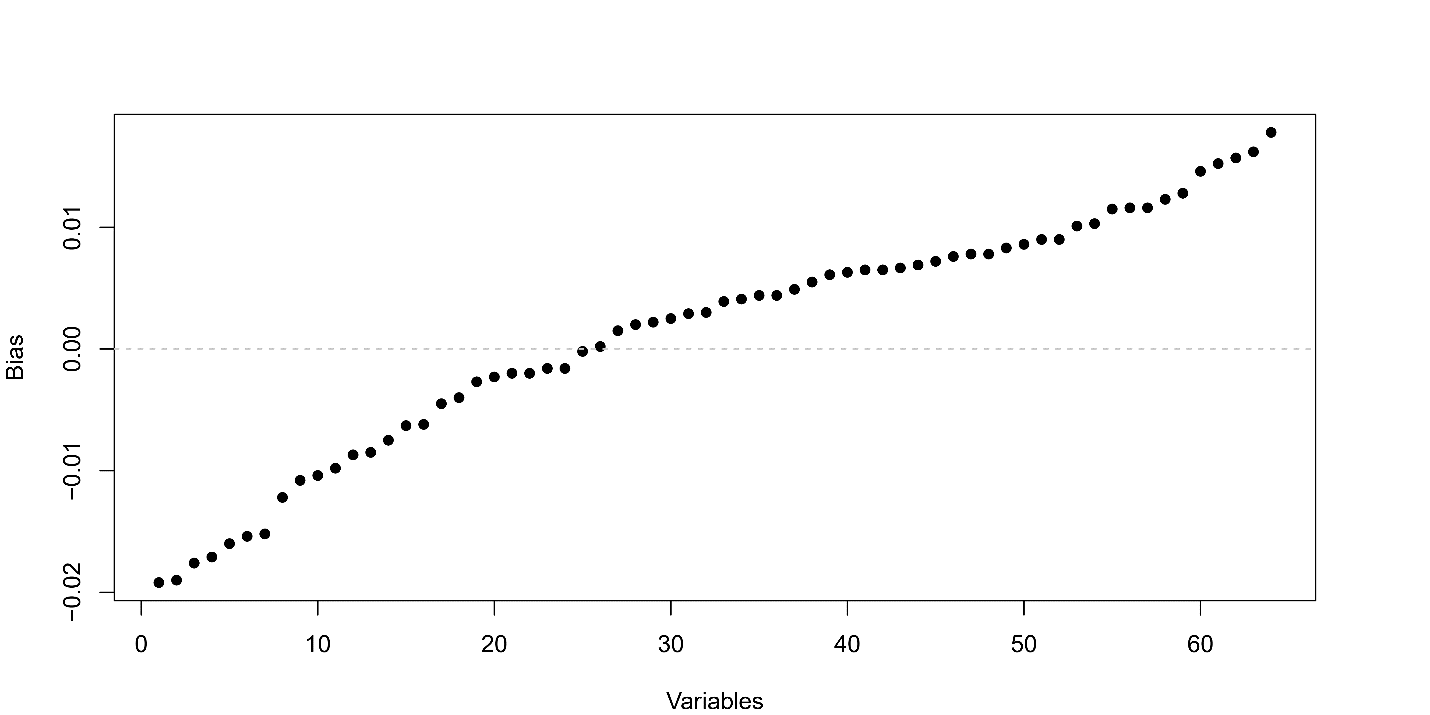
**

Supplement: Supplementary file 4 — Additional file 4. [file 12876_2024_3123_MOESM4_ESM.docx]
